# Preempting Performance Challenges: The Effects of Inoculation Messaging on Attacks to Task Self-Efficacy

**DOI:** 10.1371/journal.pone.0124886

**Published:** 2015-04-21

**Authors:** Ben Jackson, Josh Compton, Ryan Whiddett, David R. Anthony, James A. Dimmock

**Affiliations:** 1 School of Sport Science, Exercise and Health, The University of Western Australia, Perth, Australia; 2 Institute for Writing and Rhetoric, Dartmouth College, Hanover, New Hampshire, United States of America; University of Groningen, NETHERLANDS

## Abstract

Although inoculation messages have been shown to be effective for inducing resistance to counter-attitudinal attacks, researchers have devoted relatively little attention toward studying the way in which inoculation theory principles might support challenges to psychological phenomena other than attitudes (e.g., self-efficacy). Prior to completing a physical (i.e., balance) task, undergraduates (*N* = 127, *M_age_* = 19.20, *SD* = 2.16) were randomly assigned to receive either a control or inoculation message, and reported their confidence in their ability regarding the upcoming task. During the task, a confederate provided standardized negative feedback to all participants regarding their performance, and following the completion of the task, participants again reported their self-efficacy along with measures assessing in-task processes. Findings supported the viability of efficacy inoculation; controlling for pre-task self-efficacy, task performance, and relevant psycho-social variables (e.g., resilience, self-confidence robustness), participants in the inoculation condition reported greater confidence in their ability (i.e., task self-efficacy) than those in the control condition at post-task. Relative to those in the inoculation condition, participants in the control condition also experienced greater concentration disruption and self-presentation concerns during the task.

## Introduction

Psychologists, marketers, and communication theorists often utilize persuasion techniques to stimulate desired decision-making and behavioral processes, or to encourage attitude formation [1]. There are instances, however, when individuals may *already* hold a desired attitude toward a concept, and in such cases, it may be most pertinent to implement strategies that enable those individuals to withstand future attacks to that attitude. It is well documented that our attitudes can fluctuate [2]. Health-enhancing/-protective attitudes, for instance (e.g., “fast food is bad”), are regularly subject to persuasory challenges (e.g., seeing a high profile athlete endorse a fast food restaurant), potentially causing us to re-evaluate our beliefs and adopt a less desirable position (e.g., “maybe fast food isn’t so bad after all”). In light of this notion of attitude fragility, empirical attention has been directed toward identifying strategies that are effective in promoting individuals’ ability to resist persuasory attempts and maintain their attitudes in the face of challenges. In this investigation, we focus our attention on one such resistance-based framework that has received substantial scrutiny, namely *inoculation theory* [3,4].

Guided by the principles that underlie medical immunization, inoculation theory was developed out of pioneering work in which the resistance-inducing effects associated with two-sided messages were demonstrated [5]. Specifically, in medical immunization, weakened forms of a virus (i.e., vaccines) are injected into a host, in order to stimulate immune system adaptations that render the host resistant to future, stronger attacks from that virus. With respect to resisting counter-attitudinal ‘attacks’, McGuire [3,4] asserted that messages containing weakened arguments against an established attitude could enable individuals to develop resistance against stronger attacks that they may encounter to that attitude in the future. It was theorized, therefore, that inoculation may act as a preventive strategy that could bolster individuals’ resistance to the various counter-attitudinal (e.g., social, media, and/or interpersonal) influences with which they may be confronted. Moreover, McGuire [6] contended that inoculation might serve as an effective method for protecting behavior patterns. For example, by enabling adolescents to maintain an anti-smoking attitude, inoculation has the potential to display a downstream influence in terms of helping message recipients to resist pressures to smoke cigarettes [7].

Early inoculation studies targeted “cultural truisms”; that is, beliefs that were so widely accepted that they would be unlikely to have been attacked (e.g., the benefits of health screening). More recently, though, attention has been directed toward issues that may display greater intra- and inter-individual variability. For instance, inoculation principles have been utilized in relation to attitudes regarding smoking [7], alcohol consumption [8], credit card ownership [9], political debates [10], and academic plagiarism [11]. Indeed, in a recent meta-analysis [12], in which the authors synthesized over 50 empirical reports, it was concluded that inoculation messages were superior to control and supportive-only messages in conferring attitude resistance. In line with McGuire’s [3,4] original assertions, therefore, inoculation appears to represent a potent treatment that is effective in protecting one’s beliefs from typically-occurring attacks [13,14,15].

As well as testing the utility of McGuire’s [3,4] model, investigators have also provided insight into the elements that are required within/from an inoculation treatment in order to stimulate resistance to future attacks. The first of these elements is termed *threat*, and represents an awareness that one’s position on an issue may be vulnerable and subject to attacks. Typically, threat is evoked through the provision of forewarning within an inoculation treatment; that is, an explicit statement notifying message recipients that their attitudes are susceptible to change and may be challenged [16]. The second common element within inoculation treatments is referred to as *refutational preemption*. It is within this component of the inoculation message that recipients are often provided with specific content that can be used to combat subsequent attacks to their attitude. In a typical inoculation message, refutational material is presented following the provision of weakened arguments against one’s attitudes (i.e., counterarguments). That is, individuals are presented with a selection of hypothetical/possible attacks to their attitude, before being provided with refutational material that conveys information designed to overcome those attacks. In early research, this process was sometimes ‘active’ in nature, whereby participants were invited to formulate their own refutations [17]; however, more contemporary inoculation research typically uses pre-formulated (i.e., ‘passive’) refutations. Not only does this passive approach appear to elicit resistance, it also enables researchers and practitioners to devise standardized and easily-administered treatments.

Despite our knowledge about the utility of inoculation theory, and the components that constitute an effective inoculation treatment, there are a number of ways in which the inoculation literature may be advanced. We sought to address two of these issues in the present investigation by (a) extending inoculation treatments beyond attitudinal constructs, with a specific emphasis on task self-efficacy, and (b) identifying the way in which efficacy inoculation may act on a range of in-task evaluative processes. There has been a consistent focus on attitudinal inoculation in light of the way in which the theory was originally framed, which is appropriate given the frequency with which our attitudes are challenged, as well as their susceptibility to change. That said, attitudes are just one of a host of psycho-social constructs that have implications for behavioral functioning and that are subject to attack [18]. To date, the limited attention devoted to self-efficacy in inoculation has been directed toward its potential as a moderator variable [19], or as a mechanism that may be implicated in strengthening attitude resilience (e.g., coping-related efficacy beliefs reflecting individuals’ confidence in their ability to protect an attitude) [20]. With that in mind, we sought to extend the scope of existing research by investigating whether inoculation treatments can successfully confer resistance to attacks to one’s *task self-efficacy*.

### Self-efficacy Inoculation

Self-efficacy represents an individual’s confidence in his/her ability within a specific domain [21], and a high level of confidence in one’s ability in a given setting coincides with adaptive motivational and functional outcomes, including elevated academic achievement [22] and work performance [23], as well as heightened sporting [24] and musical [25] accomplishment. Importantly, although self-efficacy has received scrutiny largely due its predictive import, this construct has also attracted scholarly interest as it is recognized that individuals’ confidence beliefs are *dynamic and malleable*. Indeed, Bandura [21] detailed the means through which self-efficacy may be augmented (e.g., mastery achievements, positive verbal persuasion), and numerous investigators have utilized theory-driven strategies to successfully increase individuals’ confidence in their ability [26,27,28]. Although researchers have typically used Bandura’s proposals in order to bolster self-efficacy beliefs, the antecedents that Bandura described also emphasize the ways through which individuals’ confidence in their ability may be undermined, or ‘attacked’ (e.g., through poor performance, receiving negative feedback, undesirable emotional states). It has been demonstrated, for example, that the provision of negative performance feedback may instill self-doubt [29], and Bandura [30,31] described how the interpretation of anxiety and stress as a threat serves to reduce self-efficacy. This notion, that self-efficacy beliefs may be weakened in the face of challenges, was captured by Bandura [30], who noted that, if individuals “discover something that appears intimidating about the undertaking or suggests limitations to their mode of coping, they register a decline in self-efficaciousness” (p.125–126).

Although the malleability of individuals’ confidence in their ability is well established, strategies for making existing self-efficacy beliefs more resilient have received comparatively little attention. That is, relative to research that has focused on increasing individuals’ self-efficacy, investigators have devoted much less attention toward identifying strategies that may help self-efficacious individuals *remain* confident in their ability in the face of challenges. That is not to say that we have no understanding regarding self-efficacy resilience; indeed, there are personal characteristics that have been shown to promote more robust self-perceptions (e.g., resilience, self-confidence robustness). For example, Beattie and colleagues [32] presented a measure of self-confidence robustness, an individual difference variable that was shown to be associated with more stable appraisals regarding one’s confidence in one’s ability. Similarly, a strong sense of self-efficacy in and of itself has been shown to protect individuals against challenges to their confidence. For example, Nease, Mudgett, and Quiñones [33] reported that highly self-efficacious individuals may be less likely to accept negative performance feedback in an effort to protect their sense of self. Aside from these personal characteristics, though, relatively little attention has been devoted to exploring how *external* strategies (e.g., messages utilizing inoculation principles) can be used as a scaffold to create self-efficacy beliefs that are resistant to challenges.

In light of the challenges that individuals may encounter to their self-efficacy, our primary aim was to determine whether a theory-derived inoculation treatment could enable individuals to withstand attacks to their self-efficacy (i.e., stimuli that would, theoretically-speaking, be expected to negatively impact one’s confidence in one’s ability). Specifically, by providing challenges to self-efficacy during the performance of a novel physical (i.e., balance) task, and controlling for pre-task self-efficacy perceptions (as well as a range of theoretically-relevant background variables), we hypothesized that individuals who received an inoculation treatment (relative to a message containing efficacy-supportive information only) would report greater confidence in their ability at post-task. In addition, given that favorable self-efficacy perceptions underpin elevated performance goals [29,34], we also anticipated that participants in the inoculation (relative to control) condition would select a longer performance time when given the opportunity to participate in a subsequent (bogus) trial at the end of the balance task. It is worth noting, however, we did not anticipate that performance on the balance task itself would differ across conditions. That is, our treatment was designed not with the aim of inducing between-group differences on pre-task self-efficacy perceptions, and, we would only expect task performance differences as a function of between-group differences on pre-task self-efficacy.

### Efficacy Inoculation and In-task Processes

Aside from broadening the scope of inoculation research beyond attitude protection, we also requested participants to report (following the task) on a range of in-task processes upon which self-efficacy inoculation may act. Within the last decade, inoculation researchers have directed increased attention toward identifying the psychological processes that are implicated in inoculation effects, and have demonstrated that inoculation treatments may (a) increase issue involvement [35] and attitude accessibility [36], (b) elicit resistance-enhancing affective responses [19], (c) trigger counter-arguing processes [37], and (d) promote greater certainty about one’s ability to defend one’s attitude [20]. Notwithstanding these findings, given that we were applying inoculation principles to a new target construct, we aimed to consider how these inoculation treatments may act on relevant in-task evaluative processes [38]. With that in mind, we drew from the extant inoculation and self-efficacy literatures in order to determine the in-task processes upon which an efficacy-based inoculation treatment may operate.

Prior to the balance task, all participants were informed that their performance would be observed and assessed by an independent expert who may provide feedback regarding their performance. This independent expert was in fact a confederate who delivered standardized negative feedback to all participants (irrespective of their performance on the task and the condition to which they were assigned). More information is presented on this issue in the method section. In evaluative scenarios such as these, individuals are susceptible to experiencing *self-presentation concerns* regarding the impression that they make on others [39], which, in this case, took the form of the confederate ‘evaluator’. The provision of refutational preemptions regarding the presence and impact of this evaluator, however, may partially alleviate participants’ in-task self-presentational concerns regarding the evaluator’s impression of them [20]. Given that there is evidence of an inverse relationship between self-presentation concerns and resultant self-efficacy [40,41], we hypothesized that those in the inoculation (relative to those in the control) condition may experience less intense self-presentational concerns during the task.

Second, we assessed participants’ perceptions regarding the *impact of the evaluator’s feedback* on their task performance. Standardized feedback can be received and appraised by individuals in markedly different ways [42], and it is possible that forewarning (and the provision of refutational material) in relation to the likelihood of critical evaluator comments may lessen the potentially destructive impact of that feedback in the eyes of participants. For example, should inoculated participants (a) ultimately receive negative feedback during the task that was actually *less* critical than they had prepared themselves to receive, or (b) be in a better position to fully process and reflect upon the negative feedback, then they may be able to interpret that feedback in a relatively more positive light than control participants, who were unprepared for any such feedback [42,43]. Baron [29] demonstrated that the receipt of destructive feedback accounts for maladaptive affective responses and lowered self-efficacy; however, the severity of such a reaction likely depends on the extent to which that feedback is perceived by the recipient to be destructive or not [42]. We hypothesized, therefore, that the elements of threat and refutational preemption may enable inoculated (relative to control) participants to appraise the evaluator’s comments in a less disruptive light.

Our third in-task process targeted the extent to which inoculated (versus control) participants would experience *concentration disruption* as a result of challenges they encountered during the task. Guided by Bandura’s [21] theorized self-efficacy antecedents, inoculated participants were notified of three potential attacks to their confidence that may occur during the task. As outlined previously, the first of these attacks reflected the potential receipt of negative feedback (i.e., verbal persuasion) from the evaluator, while the remaining ‘attacks’ that were discussed included the potential for performance errors and the experience of adverse emotional states (e.g., nerves and anxiety) during the task. Having been informed that these attacks may occur, we hypothesized that inoculated (relative to control) participants would report lower concentration disruption when these challenges actually arose. Concentration disruption is recognized as an aversive process [44,45] that aligns inversely with self-efficacy via the experience of undesirable emotional states. Our final in-task process related to the notion of *feedback acceptance*. Specifically, as a result of being made aware that they may receive criticism from the evaluator, we felt it was possible that inoculated participants may pre-determine that they would simply ignore or not accept the evaluator’s feedback [33]. From a conceptual perspective, negative feedback would be expected to lower one’s self-efficacy [21]; however, this may not occur in cases where feedback is ignored or not accepted [42]. Accordingly, rather than (or as well as) acting upon other putative in-task processes, it was possible that the inoculation treatment may exert its influence by simply enabling participants to implement avoidance strategies that discredit the negative impact of the verbal attack.

In sum, our primary aim was to examine the applicability of inoculation treatments outside of attitudinal constructs, with a specific emphasis on the use of this strategy in helping individuals withstand challenges to their confidence in their ability (i.e., task self-efficacy) during a performance scenario. Within this overarching purpose, our supplementary aim was to identify (some of) the mechanistic processes (i.e., self-presentation concerns, perceptions of feedback, concentration disruption, feedback acceptance) that may underpin any emergent effect of task self-efficacy inoculation.

## Method

### Participants

The initial participant pool consisted of 184 undergraduates majoring in Kinesiology at the lead author’s institution and participated in return for course credit. Given that the hypothesized effects relied on participants attending to the content of the inoculation or control message, all participants completed a brief recall test immediately after reading the message (see following section for procedural details). Specifically, participants were asked to recall seven pieces of memorable information (the exact same seven pieces of information were included in both messages), and those who scored below the mid-point (i.e., three or fewer correct answers) were dropped from further analyses. After screening individuals using this recall variable, the final sample comprised 127 undergraduates (*M*_*age*_ = 19.20, *SD* = 2.16, 59 males, 68 females). Although this process helped us ensure that all those who were included in the final analyses had attended sufficiently to the message (and had not simply guessed one or two correct answers on the recall test), we recognize that this screening procedure did result in a loss of power, and that the mid-point cut-off may appear somewhat arbitrary. With that in mind, we present an appendix to our results section in which we report on the exact same main analysis procedure, but with the analyses performed on the entire sample (i.e., irrespective of recall score).

### Procedure

This research was approved by the Human Research Ethics Office at The University of Western Australia (approval RA/4/1/6138). Undergraduates were informed in a lecture that they could receive course credit for participating in a study on balance. To avoid arousing suspicion as to the true nature of the study, all prospective participants were informed that the testing was taking place at the request of an employee from a regional sporting institute, who had requested that the investigators obtain normative data in order to enable comparisons between an ‘athletic’ undergraduate population (i.e., kinesiology majors) and a new cohort of young elite athletes within the institute. All undergraduates who registered their interest were given an information sheet and provided their written informed consent in a subsequent tutorial, before also completing background questionnaires assessing self-confidence robustness, resilience, and perceived competence at agility-based activities. At this point, participants were invited to make an individual appointment three to five weeks later in order to complete the remainder of the procedure. Participants were randomly assigned to the inoculation or control condition, and during each testing session, participants were greeted in a standardized way by a research assistant (who was the same throughout all sessions), before being seated and provided with a one-page, double-sided information sheet relating to the balance task they were to perform.

All participants received a standard introduction message in the information sheet that highlighted the nature and importance of the balance task. In addition to receiving a comprehensive description of the requirements of the task, participants were informed that the task they were to complete was “a validated and widely-used measure of functional ability”, and were also instructed that “a qualified sport scientist will rate your performance on the balance task and you will obtain an overall balance score, which is generated from a number of performance components, including reaction time, speed of movement, fluidity and consistency of movement, agility, and pressure distribution.” In order to emphasize the significance of the task, participants were also provided with bogus material informing them that “research has shown that performance on this task… is an extremely good indicator of your functional capacity and stability in older age. Research has also shown that individuals who progress to high levels in athletic pursuits score better than others on this balance task. Finally, given that this task is novel for most people, researchers have also used it as a marker of how well individuals adapt to novel tasks. People who attain a high overall balance score on their first attempt have been shown to be more intelligent and adaptive in novel situations.”

All participants were subsequently presented with material that repeated the bogus purpose of the study, and were informed that the sport scientist who would be appraising their performance was an employee of the regional sport institute specified in the purpose statement. Finally, all participants received a brief efficacy-supportive message that was derived using the theorized determinants of self-efficacy [21]. This efficacy-supportive message was designed to provide individuals with information relating to relevant mastery experiences. In particular, participants were informed that, “if you’re majoring in kinesiology then you’re likely to be adept at physical activity tasks such as this one. Previous research with male and female kinesiology students has shown that this cohort typically attains a high overall balance score. It is likely that you perform balance tasks like this every day without any problems (e.g., riding a bicycle, walking down stairs).” Following these standard sections, participants in the control condition were asked to complete the recall test and continue with the protocol. Participants in the inoculation condition, however, received additional inoculation (i.e., threat, counterarguments, refutations) material in this information sheet; a complete description of the inoculation message is provided in the following section.

Following the recall test, participants were asked to complete measures assessing pre-task tension, perceived threat, message credibility, and self-efficacy (see measures section for full descriptions of these and all other measures). Participants were then escorted to an adjacent room and introduced to a male confederate (the same confederate was used throughout), who was described to participants as an employee of the previously-mentioned sport institute. The confederate was dressed in clothing emblazoned with the institute’s logo, and participants were informed that the confederate held a doctoral degree with a specialization in biofeedback. Using a standardized script, the confederate reiterated the task instructions that had been provided previously in the information sheet; notably that participants were required to stand in the center of a platform that was fixed to the ground in front of a 1.5m x 2m projector screen. The confederate, who was sitting at a desk directly behind the participant throughout the task, also instructed participants that they were required to move their center of pressure into a series of circular red targets that would appear on the screen, while avoiding moving their center of pressure into a series of circular blue obstacles that would also appear on the screen. The confederate instructed participants that when they successfully held their center of pressure within a red target for two seconds, this target would disappear and would be replaced by another target-obstacle pairing. The activity lasted two minutes and was developed specifically for this investigation on the basis that undergraduates would have no direct prior experience with the task; no participants reported having previously performed the task or having used the balance equipment.

Approximately 45 seconds into the task, and again at approximately 90 seconds, the confederate delivered standardized negative feedback to all participants. Specifically, the confederate was instructed to select (and provide) a negative performance-related comment from a standardized list of five possible comments at each time point (i.e., “concentrate—your results are shaky,” “remember—you must avoid those blue obstacles,” “your results indicate that you’re tiring,” “your reaction time is slow,” and, “careful—your performance is looking shaky”). The confederate was asked not to provide comments that clearly misrepresented the participant’s performance at that moment in time, and was instructed to select the comment that was most suited to the participant’s task execution at the time of delivery. Immediately upon completing the task, participants were escorted out of the room by the research assistant and completed measures assessing task importance and measures assessing in-task processes. We included a single-item within the post-task questionnaire in order to check that all participants believed the confederate was credible (i.e., “I thought the sport scientist was credible”), which was scored from 1 (*not at all true*) to 7 (*very true*). We observed high average scores for participants in the inoculation (*M* = 5.42, *SD* = 1.26) and control (*M* = 5.23, *SD* = 1.25) conditions, and a one-way ANOVA revealed that inoculation and control participants did not differ in their perceptions regarding the credibility of the confederate (*F*(1, 125) = .68, *p* = .41, η^2^_p_ = .005).

Finally, having completed the post-task questionnaire, participants were instructed that they would undertake a second balance task (using the same equipment and requiring the same skills as the task they had just completed) in a forthcoming tutorial session. This information was erroneous; participants were made to believe that there was a second task solely to ensure that they had a point of reference upon which to rate their self-efficacy following the first task. Having rated their confidence regarding the second task, participants were also instructed that they were able to choose the length of their second trial in order to obtain a measure of goal striving/intended persistence for the second task. Following the completion of all trials, all participants were debriefed as a group regarding the true nature of the activity, and were informed that there was no second task.

### Experimental Manipulation

Alongside the standardized material that was presented to inoculation and control participants (i.e., introduction, task description, fictitious purpose statement, efficacy-supportive message), individuals in the inoculation group were provided with additional material derived using inoculation theory principles. In particular, participants were first provided with a forewarning in order to emphasize the potential challenges they may face during the task (i.e., to induce perceptions of threat regarding their capabilities). Individuals were told, “there will be challenges—while you may believe you have a grasp of the balance task you are about to perform, you may find the task to be difficult.” In addition to this forewarning, inoculation participants were provided with a series of counterarguments and paired (i.e., passive) refutations that targeted three potential challenges/attacks relating to the balance task (see S1 Appendix). These challenges were again derived using the theorized determinants of self-efficacy outlined by Bandura [21]. The first counterargument-refutation pairing was designed to highlight and address challenges posed by the sport scientist (i.e., confederate), and focused specifically on the potential for negative feedback (i.e., verbal persuasion). The second pairing was designed to highlight and refute challenges associated with ineffective performance, and the final pairing was designed to highlight and refute challenges associated with adverse emotional states during the task (i.e., experiencing anxiety).

## Measures

### Background variables

#### Participation in balance-based activities

Participants were asked to indicate with a ‘yes/no’ response whether they participated in a balance-based sport or activity (i.e. gymnastics, martial arts, surfing, skateboarding, or self-reported other) on a regular basis (i.e., at least once every fortnight).

#### Self-confidence robustness

An eight-item instrument was used to measure the extent to which participants felt that they were able to typically maintain their confidence in the face of challenges [32]. In line with original recommendations, participants were asked to respond to each statement while thinking about how performance may affect their confidence generally, and responses were made on a scale ranging from 1 (*strongly disagree*) to 9 (*strongly agree*). A mean score was obtained across all items (as was the case for all other non-single-item variables), and example items included, “My self-confidence is stable; it does not vary much at all,” and “my self-confidence goes up and down a lot” (reverse scored). Beattie et al. [32] presented evidence for the structural properties, convergent validity, and internal consistency of measures derived from this instrument.

#### Resilience

The six-item Brief Resilience Scale [46] was used to measure participants’ general level of resilience. Using a scale anchored at 1 (*strongly disagree*) and 5 (*strongly agree*), participants indicated the extent to which they agreed with each item (e.g. “I tend to bounce back quickly after hard times,” “it does not take me long to recover from a stressful event”). Existing work has demonstrated support for the unidimensionality, criterion validity, and reliability of measures derived from this instrument [46].

#### Perceived competence at agility-based activities

Participants reported their perceived competence at agility-based tasks using the six-item perceived competence subscale from the Intrinsic Motivation Inventory (IMI) [47]. Using a response scale ranging from 1 (*not at all true*) to 7 (*very true*), participants were asked to report the extent to which each statement (e.g. “I think I am pretty good at agility-based activities”) was true for them. Previous research has demonstrated support for the internal consistency of measures derived from this instrument [48].

#### Pre-task tension

The four-item tension subscale from the Profile of Mood States-Adolescent (POMS-A) [49] was used to measure participants’ levels of tension immediately prior to the balance task. Using a Likert scale anchored at 0 (*not at all*) to 4 (*extremely*), participants indicated the extent to which a series of descriptors (i.e., ‘panicky’, ‘anxious’, ‘worried’, ‘nervous’) were accurate in terms of how they felt at that moment in time. Previous research has demonstrated support for the psychometric properties of the POMS-A with adolescent and adult populations [49,50].

### Manipulation checks and inoculation components

#### Message credibility

We used two items to assess participants’ perceptions regarding the credibility of the (inoculation or control) message. First, using a response scale ranging from 1 (*not at all credible*) to 9 (*very credible*), participants were asked, “how credible did you find the information you received in the information sheet?” Second, using a nine-point response scale anchored at 1 (n*ot at all convincing)* and 9 (v*ery convincing*), we asked participants, “how convincing did you find the information that you received in the information sheet?”

#### Perceived threat

One item was used to assess participants’ perceptions of threat relating to the activity. Participants were asked to respond to the statement, “I view the prospect of potential challenges to my balance performance as…”, using a bipolar response scale anchored at 1 (*unlikely*) and 7 (*likely*). Given that we were testing inoculation of self-efficacy, we concluded that the typical threat scale used in attitudinal inoculation [20] would not be appropriate, as some of this scale’s bipolar adjective pairings could, we believe, be tapping into measures of self-efficacy beliefs (e.g., threatening/nonthreatening). To avoid conflating threat with self-efficacy, we used the measure described here.

#### Task importance

Perceptions of task importance were measured using the five-item effort/importance subscale from the IMI [47]. Using a response scale ranging from 1 (*not at all true*) to 7 (*very true*), participants were asked to rate how true each statement was for them (e.g. “it was important to me to do well in the balance task”). Validity and reliability evidence has been documented for this IMI subscale [51].

#### Task performance

To obtain an objective measure of task performance, we recorded the number of successful target hits that were made during the two-minute trial. A successful hit was recorded when a participant maintained his/her center of pressure within a red target for two seconds, resulting in the target disappearing and being immediately replaced by another target.

### Primary variables

#### Self-efficacy

A nine-item, non-hierarchical instrument was developed specifically for this activity in line with Bandura’s [52] instrument construction guidelines. Consistent with recommendations regarding the optimal response format for efficacy instruments [53,54], a five-point response scale anchored at 1 (*no confidence at all*) and 5 (*complete confidence*) was employed, and participants were asked to rate their confidence in their ability to carry out the various sub-skills associated with effective performance on the balance task (e.g., “adapt your posture in order to perform the task effectively,” “maintain your stability throughout the task, even in the face of difficult obstacles,” “maintain your concentration on the task at all times”). These nine items were developed through consultation with an expert (i.e., an Associate Professor in Motor Control) who had over 10 years’ experience using the balance equipment within his research program and undergraduate teaching. Participants initially completed this instrument immediately prior to undertaking their balance task (having received all instructions relating to the nature and requirements of the task), and were then asked to complete the instrument again following the first task in relation to the fictional second task. The exact same nine items were used for the second assessment; however, the instructions were modified to ensure participants’ ratings were specific to the second task.

#### Task-related intentions

Having been informed about the (bogus) second task and having reported their self-efficacy, participants were instructed that they were able to choose the length of their second trial (in 15 second intervals ranging between 15 and 120 seconds). Participants were also instructed that “the longer time that they selected, the more likely it was that their performance on the trial would be a true representation of their ability”. We included this instruction to encourage those who were confident in their ability to believe that performing a longer second trial was likely to be beneficial. In addition, and in order to provide an incentive for the second trial [21], participants were instructed that better performance on the second trial would be rewarded with more entries into a random prize draw to win an iPad (in reality, all those who participated received only one entry into the draw). The length of time that participants selected for their second trial was used as a marker of their intended persistence.

### In-task appraisals

#### Feedback acceptance

Following their first trial, participants were asked to respond to five statements assessing the extent to which they paid attention to and accepted the feedback provided by the confederate [33,55]. Example items included, “I paid attention to the sport scientist’s feedback,” and “I did not believe that the sport scientist’s feedback was accurate (reverse scored)”, and participants indicated the extent to which they agreed with each statement using a response scale that ranged from 1 (*strongly disagree*) to 5 (*strongly agree*), where higher scores indicated greater feedback acceptance [33].

#### Self-presentation concerns

Four items were developed in line with existing self-presentation and social anxiety measures [56], in order to assess the extent to which participants were concerned about how they were viewed/evaluated by the sport scientist. Using a seven-point response scale anchored at 1 (*not at all true*) and 7 (*very true*), participants were asked to respond to a series of statements, specifically, “I was worried about embarrassing myself,” “I was concerned during the task about what the sport scientist thought about me,” “I felt anxious when the sport scientist told me I was making mistakes,” and, “I was concerned about looking uncoordinated.”

#### Concentration disruption

Three items were used to assess the extent to which participants felt that their concentration was disrupted as a result of challenges faced during the task. Participants were asked to report how true each statement was for them using a response scale ranging from 1 (*not at all true*) to 7 (*very true*), where higher scores indicated greater concentration disruption. The items were based on the three primary challenges that individuals were likely to face during the task; that is, the challenges about which individuals in the inoculation condition were forewarned. The items were, “my concentration was disrupted when the sport scientist told me I was making mistakes,” “my concentration was disrupted when I felt I was performing poorly,” and, “my nerves disrupted my concentration.”

#### Perceived impact of confederate on performance

Having completed the balance task, participants responded to a single item assessing their perception about the impact of the confederate on their performance. Specifically, we asked participants, “what impact do you feel the sport scientist had on your performance during the balance task?” A response scale anchored at -3 (*strong negative impact*), 0 (*no impact at all*), and 3 (*strong positive impact*) was used.

## Results

### Background Analyses

A preliminary MANOVA exploring potential differences on background variables (i.e., self-confidence robustness, resilience, perceived competence at agility-based tasks, pre-task tension) revealed a nonsignificant multivariate effect for experimental condition (*F*(4, 122) = .97, *p* = .42, η^2^_p_ = .03, λ = .97), indicating that individuals in the message conditions did not display underlying differences on these background variables (see Table 1 for descriptive data according to condition, and Table 2 for descriptive data, internal consistencies, and zero-order correlations across the entire sample). Additionally, 29 participants reported engaging in a balance-based activity; however, a chi-square analysis revealed no significant effect for experimental group, χ^2^(1) = .52, *p* = .47. We had no a priori expectation regarding the relative impact of the control/inoculation messages on pre-task self-efficacy, and so we did not form a hypothesis regarding between-condition differences on this variable (see Table 1 for mean data by condition). For exploratory purposes, however, we explored the potential for such differences using a one-way, between-subjects ANOVA, which revealed no significant differences existed between inoculation and control participants (*F*(1, 125) = .20, *p* = .66, η^2^_p_ = .002).

**Table 1:** Descriptive statistics according to condition.

	Inoculation (*n* = 67)		Control (*n* = 60)	
	*M*	*SD*	*M*	*SD*
*Background variables*				
Self-confidence robustness	4.83	1.16	4.65	1.17
Resilience	3.44	.68	3.57	.62
Perceived competence	4.60	1.17	4.65	1.09
Pre-task tension	1.11	.76	1.09	.79
*Manipulation checks & inoculation components*				
Message credibility	7.17	1.06	6.98	1.07
Perceived threat	4.43	1.36	4.00	1.34
Task importance	5.52	.91	5.38	.81
Task performance	10.67	3.60	11.07	3.32
*Primary variables*				
Self-efficacy (pre-task)	3.34	.48	3.30	.47
Self-efficacy (post-task)	3.53	.54	3.30	.58
Task 2 intended length	99.18	27.87	94.75	31.30
*In-task perceptions*				
Feedback acceptance	3.64	.80	3.54	.74
Self-presentation concerns	3.37	1.35	3.97	1.37
Concentration disruption	3.31	1.19	3.86	1.24
Confederate impact	.22	1.15	-.28	1.03

*Note*. Self-confidence robustness and message credibility measured 1 to 9, resilience and self-efficacy 1 to 5, and perceived competence and task importance 1 to 7, where higher scores represented more favorable perceptions. Tension measured 0 to 4, and threat measured 1 to 7, where higher scores represented greater perceived tension/threat, and feedback acceptance 1 to 5, where higher scores indicated greater acceptance. Self-presentation concerns and concentration disruption measured 1 to 7, where higher scores represented greater concerns/disruption. Task performance measured in terms of number of targets hit, and task 2 intended length could range from 15 to 120 sec. Confederate impact ranged -3 to 3, where scores < 0 indicated a negative impact and scores > 0 indicated a positive impact.

**Table 2:** Descriptive data, internal consistency, and zero-order correlations for all variables across the entire sample.

Variable	*M* (*SD*)	Skew.	Kurt.	IC	2	3	4	5	6	7	8	9	10	11	12	13	14	15
1. Self-confidence robustness	4.74 (1.17)	.27	-.35	.84	.46	.23	-.20	-.02	-.25	-.05	.02	.41	.42	.01	-.21	-.31	-.23	.06
2. Resilience	3.50 (.65)	-.21	-.07	.84	-	.07	-.23	.06	-.07	.01	.03	.17	.23	.03	-.05	-.27	-.33	.07
3. Perceived competence	4.62 (1.13)	-.78	.66	.94		-	-.22	-.01	-.23	.01	.28	.49	.33	.16	-.06	-.09	-.06	.14
4. Pre-task tension	1.10 (.79)	1.06	.80	.89			-	-.19	.29	-.04	-.10	-.37	-.21	.04	.07	.38	.29	-.10
5. Message credibility	7.08 (1.06)	-.10	-.36	.83				-	-.01	.23	.12	.12	-.08	-.18	.11	.06	-.04	.14
6. Perceived threat	4.23 (1.36)	.02	-.46	—					-	.11	-.10	-.32	-.29	-.07	-.03	.04	.01	.06
7. Task importance	5.45 (.86)	-.09	-.41	.81						-	.13	.16	.13	.04	.04	-.07	-.14	.16
8. Task performance	10.86 (3.46)	.02	-.63	—							-	.17	.08	.12	-.10	.10	.01	.18
9. Self-efficacy (pre-task)	3.32 (.48)	.20	.80	.83								-	.61	-.04	-.05	-.25	-.20	.12
10. Self-efficacy (post-task)	3.42 (.57)	-.07	-.33	.89									-	.15	-.09	-.44	-.30	.18
11. Task 2 intended length	97.09 (29.51)	-.99	-.25	—										-	.12	.08	-.02	.08
12. Feedback acceptance	3.59 (.77)	-.71	.79	.81											-	.19	.07	.21
13. Self-presentation concerns	3.65 (1.39)	.07	-.91	.84												-	.62	-.25
14. Concentration disruption	3.57 (1.24)	.26	-.19	.78													-	-.31
15. Confederate impact	-.02 (1.12)	.41	-.62	—														-

*Note*. IC = internal consistency (for all non-single-item scales). All IC values represent alpha coefficients, excluding message credibility, which was computed from a two-item scale and so was estimated using Spearman-Brown coefficient *ρ*.

> |. 18 | = *p* <.05; > |. 23 | = *p* <.01; > |. 31 | = *p* <.001.

### Manipulation Checks and Inoculation Components

A MANOVA was used to examine between-group differences on the variables that may have influenced post-task self-efficacy (i.e., task performance) and/or that were viewed as necessary preconditions for inoculation to occur (i.e., perceived threat, task importance, message credibility). We observed a nonsignificant multivariate effect (*F*(4, 122) = 1.22, *p* = .31, η^2^_p_ = .04, λ = .96), demonstrating that participants in each condition did not differ on these variables (see Table 1).

### Main Analysis

In our primary analyses, we used a MANCOVA to investigate between-condition differences on (a) post-task self-efficacy, and (b) the length of time that participants selected for their (bogus) second trial. Accordingly, when examining univariate significance we used an adjusted criterion (i.e., α = .025) in light of the number of dependent variables within the analysis. We explored differences on these two dependent variables while controlling for the potential effect of background variables (i.e., self-confidence robustness, resilience, perceived competence at agility-based tasks, pre-task tension), as well as participants’ pre-task self-efficacy and task performance score. A significant multivariate effect emerged (*F*(2, 118) = 3.58, *p* = .03, η^2^_p_ = .06, λ = .94), which was accounted for by differences on post-task self-efficacy (*F*(1, 119) = 6.88, *p* = .01, η^2^_p_ = .06), but not by the length of time that participants selected for their second trial (*F*(1, 119) = 1.18, *p* = .28, η^2^_p_ = .01). Specifically, when controlling for background variables, as well as participants’ task performance and initial (i.e., pre-task) confidence in their ability, inoculated participants reported greater confidence in their ability at post-task than their counterparts in the control condition (see Table 1). For coverage of the results (and interpretation) of these analyses when performed on the entire sample (i.e., irrespective of recall scores), see S2 Appendix.

### Supplementary analyses

Although our primary analyses demonstrated that inoculation participants reported greater self-efficacy than their control counterparts at post-task, we felt that it may be worthwhile to conduct follow-up analyses in order to examine the specific nature of change (in self-efficacy) over time for inoculation and control participants. Accordingly, we performed a two-way ANOVA in which time (i.e., pre-, post-task) and condition (i.e., inoculation, control) were treated as independent factors, self-efficacy was entered as the dependent variable, and in which all covariates specified previously were retained (with the exclusion of pre-task self-efficacy). We observed no main effect for time (*F*(1, 120) = 0.64, *p* = .43, η^2^_p_ = .005) or condition (*F*(1, 120) = 3.21, *p* = .08, η^2^_p_ = .03); however, as would be expected in light of the finding reported above, a significant time-by-condition interaction did emerge (*F*(1, 120) = 5.74, *p* = .02, η^2^_p_ = .05). When probing for differences over time within the control condition only (using a paired-samples t-test), we observed that control participants’ self-efficacy did not change over time from pre- to post-task (*t*(59) = 0.13, *p* = .90; see Table 1 for summary data). For those in the inoculation condition, however, we observed a significant increase in self-efficacy from pre- to post-task (*t*(66) = -3.30, *p* = .002; see Table 1).

### Analysis of In-task Perceptions

In this instance, we again used a MANCOVA to investigate between-condition differences on (a) feedback acceptance, (b) self-presentation concerns, (c) concentration disruption, and (d) participants’ perceptions regarding the impact of the confederate on their performance. The significance level for univariate follow-ups was set at. 0125, and we included the same range of covariates as in our primary analysis. A significant multivariate effect emerged (*F*(4, 116) = 3.81, *p* = .006, η^2^_p_ = .12, λ = .88), which was accounted for by differences on self-presentation concerns (*F*(1, 119) = 7.02, *p* = .009, η^2^_p_ = .06), concentration disruption (*F*(1, 119) = 9.17, *p* = .003, η^2^_p_ = .07), and perceptions regarding the impact of the confederate (*F*(1, 119) = 8.16, *p* = .005, η^2^_p_ = .06). Relative to those in the inoculation group, control participants experienced greater self-presentation concerns during the task, and reported that their concentration was disrupted to a greater extent by adverse events during the task (e.g., when receiving criticism; see Table 1 for descriptive data). Also, although control participants reported that the confederate had a weak negative impact on their task performance, those in the inoculation condition felt that the confederate had a weak positive impact on their performance (see Table 1). Participants in the two conditions did not differ in terms of the extent to which they accepted the confederate’s feedback (*F*(1, 119) = .91, *p* = .34, η^2^_p_ = .01).

Our final analytic procedure was conducted with the aim of examining the extent to which these four in-task perceptions supported indirect relations between treatment (i.e., inoculation or control) assignment and participants’ post-task self-efficacy. To do so, we utilized Preacher and Hayes’ INDIRECT SPSS macro with bootstrapping for multiple mediation [57]. We entered treatment (coded 1 for inoculation and 0 for control) as the independent variable (IV), all in-task perceptions as proposed mediators (M), and post-task self-efficacy as the dependent variable (DV). In line with findings reported above, analyses of IV → M pathways revealed significant effects for treatment in relation to self-presentation concerns (estimate = -.59, *SE* = .24, *p* = .02), concentration disruption (estimate = -.55, *SE* = .22, *p* = .01), and perceptions regarding the impact of the confederate on their performance (estimate = .51, *SE* = .19, *p* = .01). In terms of M → DV pathways, we observed a significant effect between self-presentation concerns and post-task self-efficacy (estimate = -.19, *SE* = .04, *p* = .001), which indicated that greater self-presentation concerns were related to lower post-task self-efficacy (no other significant M → DV pathways emerged). The confidence interval for the bootstrapped total indirect effect from treatment to post-task self-efficacy (through self-presentation concerns) excluded zero (estimate = .11, SE = .05, 95% bias corrected confidence interval. 02,. 24), and the overall normal theory test associated with the indirect effect was significant (z = 2.13, *p* = .03). Taken together, these analyses revealed that the inoculation treatment elicited (among other things) lower self-presentation concerns, which in turn promoted more favorable post-task self-efficacy perceptions.

## Discussion

Sustained research spanning the last 50 years has established that inoculation is an effective method for bolstering resistance to counter-attitudinal attacks [12]. However, scholars have devoted relatively little attention toward identifying whether, and how, inoculation principles might support attacks to other psychological constructs. With that in mind, we drew from McGuire’s [6] framework in order to devise an inoculation treatment that may enable individuals to withstand attacks to their confidence in their ability to perform a task (i.e., task self-efficacy). Importantly, not only is task self-efficacy a prepotent predictor of motivational and achievement-related outcomes [21], it is also malleable and can be revised in both an upward [26,27] and downward [29] direction. With an emphasis on the potential for self-efficacy beliefs to diminish in the face of adverse environmental (e.g., negative performance feedback) and experiential (e.g., performing poorly, feeling anxious) influences, we sought to determine whether it was possible to inoculate against the damaging (i.e., efficacy-reducing) effects of these ‘attacks’.

Our primary analyses addressed the hypothesis that individuals who were exposed to an inoculation treatment—prior to experiencing self-efficacy-related ‘attacks’ during a standardized physical (i.e., balance) task—would report greater post-task self-efficacy in comparison to individuals in a control condition (while controlling for pre-task self-efficacy). We observed support for this hypothesis insofar as inoculated participants did indeed report greater confidence in their ability following their performance in the trial. In seeking to maximize the robustness of this finding, we also demonstrated that participants in the inoculation and control conditions did not differ on a range of theoretically-relevant background and pre-task variables. Moreover, we controlled for participants’ scores on these and other relevant (e.g., task performance) indices when exploring post-task self-efficacy differences. In terms of conceptual and applied relevance, this finding not only advances our understanding of the scope of inoculation effects (i.e., beyond the protection of attitudes), but also indicates that inoculation treatments may represent an effective and practical approach for supporting individuals’ self-efficacy perceptions.

Although these findings broadly supported our primary hypothesis, it is important to reflect on the exact pattern of pre-to-post-task change in self-efficacy that we observed for inoculation and control participants. According to the extant inoculation literature [12,14,15], we would anticipate that while participants in the control condition would register a decline in self-efficacy from pre- to post-task (i.e., as a result of being unprepared for the efficacy-reducing ‘attacks’ to which they were exposed), those in the inoculation condition would be insulated against such losses and would display stable task self-efficacy appraisals. Our supplementary analyses demonstrated, however, that participants in the inoculation condition actually reported *greater* confidence in their ability at post- (relative to pre-) task, and that, rather than being impaired, self-efficacy beliefs for those in the control condition were unchanged over time.

The stability of self-efficacy perceptions among control participants may have been caused, in part, by the strength of their pre-task self-efficacy perceptions. As has been demonstrated previously [21,33], a strong sense of self-efficacy in itself may give rise to resilience. Given that control participants reported moderate-to-high levels of confidence in their ability prior to the activity (see Table 1), it is possible that their pre-task confidence might have buffered against the attacks to self-efficacy, and may have contributed to the consistency in self-efficacy that was observed over time. On a separate note, descriptive data also showed that control participants were, on the whole, a relatively resilient cohort who possessed favorable perceptions regarding their competence at agility-based tasks, which may have further protected these individuals against confidence decrements. An examination of control participants’ scores on the range of in-task processes that we assessed (i.e., self-presentation concerns, concentration disruption, impact of confederate feedback) may provide some support for this assertion. In particular, although those in the control condition reported less desirable scores relative to their inoculated counterparts, they did not (in absolute terms) report markedly unfavorable perceptions on any of these indices (see Table 1), indicating that the activity may not have been viewed as sufficiently challenging in order to elicit reductions to their self-efficacy. Finally, it is worth noting that, despite not being forewarned regarding the challenges that they would encounter during the task, control participants still reported moderate levels of threat prior to their participation in the activity. In line with inoculation theory tenets [6], it is possible that this degree of threat may have triggered some level of preparatory cognitive processing, which may have aided in supporting individuals’ responses to challenges during the activity.

It is also necessary to reflect upon (and contrast this finding against) the pattern of change that was apparent for inoculation participants. Specifically, despite reporting comparable levels of resilience, perceived competence, pre-task self-efficacy, and threat (in comparison to control participants), and recording no difference in terms of actual task performance, inoculated participants displayed a significant increase in self-efficacy from pre- to post-task. One plausible explanation for this finding is that the attacks that were highlighted in the inoculation message—and participants’ subsequent elaboration on those and other potential attacks [37]—may have led individuals to somewhat overestimate the severity of the challenges that awaited them during the task. Accordingly, if inoculated individuals perceived that they were able to cope adequately with the challenges that they faced, then (given the absence of objective performance feedback following the task) they may have interpreted this as a sign that they were actually more capable than they initially believed, thus resulting in an upward revision of their self-efficacy following the task [58].

The in-task processes that we examined provided some support for this notion, insofar as inoculated (relative to control) participants retrospectively reported more favorable experiences *during* the performance of the task (i.e., lower in-task self-presentation concerns and concentration disruption). Moreover, in comparison to those in the control condition, inoculated participants also perceived that the confederate’s feedback had a relatively positive impact on their performance. When interpreted as a vehicle for development and growth, negative feedback can be viewed as a means to stimulate performance improvement [42], and this evaluative response may therefore have further contributed to the self-efficacy increase that was observed in the inoculation condition. Taken together, it is possible that the preparatory processes that are triggered by inoculation messages, along with the more favorable in-task experiences that inoculated participants reported (relative to those in the control condition), may have encouraged inoculated participants to derive confidence from their engagement in the activity (e.g., “that wasn’t so bad”, “I coped with that pretty well”). In sum, despite a somewhat unexpected inoculation effect, the *relative* between-group differences that we observed provide support for the potency of inoculation in terms of shaping the way in which individuals’ self-efficacy appraisals change (or not) in the face of theory-driven attacks.

Prior to considering design strengths, limitations, and accompanying future research directions, it is important that we address two noteworthy nonsignificant effects that we observed. First, despite detecting significant between-group differences on three of our four theorized in-task processes, inoculation and control participants did not differ in terms of the extent to which they attended to/accepted the confederate’s feedback. We hypothesized that an inoculation treatment might stimulate the use of a heuristic based on discrediting/ignoring the confederate’s feedback. From a practical perspective, however, the absence of any between-group difference (see Table 1) on this variable may hold important implications for the utility of self-efficacy-based inoculation treatments. In particular, although receiving and internalizing negative feedback may be damaging to one’s sense of self in the short term, individuals may actually benefit in the longer term by appraising, processing, and learning from the negative feedback that they receive [42]. Consider, for example, an athlete who retains his/her confidence in his/her ability by simply blocking out all negative feedback provided by his/her coach. This strategy may be effective in an acute sense by limiting the damaging effect of disparaging comments, but over time, the athlete’s progression may be marginalized as s/he fails to attend to constructive negative feedback that is designed to correct technical flaws and facilitate skill development. To return to the medical analogy to which inoculation is tied, this would be similar to individuals avoiding challenges to health (e.g., avoiding public places where they might contract illness) as a protective mechanism instead of meeting the challenges (e.g., through inoculation, or exposure to weakened viruses). It was noteworthy therefore, that inoculated participants in this study reported greater post-task self-efficacy *despite* attending to and accepting the confederate’s feedback to the same degree as those who were not forewarned regarding the likelihood of negative feedback. In that sense, inoculation treatments may be effective for protecting/promoting self-efficacy in the face of negative feedback, and importantly, this may occur without eliciting undesirable side effects that may arise when negative feedback is simply blocked out.

Second, although we observed differences in terms of participants’ post-task self-efficacy, this did not translate into significant between-group differences regarding the length of time that participants selected for the (bogus) second trial. From a self-efficacy theory perspective [21], we would anticipate that greater post-task self-efficacy would account for elevated performance goals in subsequent trials. However, Bandura [21] also outlined a number of issues that may induce discordance between efficacy appraisals and goal processes, and one or more of these factors may have been responsible for the nonsignificant effect in this investigation. Specifically, although we offered a potential reward for performance in the second trial (i.e., entries into a prize draw), this may not have provided sufficient incentive to act, and may have resulted in participants’ not fully utilizing their perceptions of ability when determining their performance time for the second trial. Alternatively, this incentive may have actually been strong enough to induce a ceiling effect, whereby all participants—irrespective of their treatment assignment—were highly motivated to attain the reward. In future, researchers wishing to examine similar inoculation-based processes might modify our approach by offering a free-choice period following the first task in which participants are allowed to practice their balance (or other) skills for an indefinite period. In such instances, highly confident individuals would likely display enhanced persistence (i.e., practice for longer) in comparison to their inefficacious counterparts [21].

In reflecting on the strengths of this investigation, it is worth noting that our points of departure from extant inoculation research are important in three main ways. First, we explored a non-attitudinal construct—self-efficacy—as the target of inoculation efforts. To date, the integration of efficacy beliefs in inoculation scholarship has been mostly limited to treating it as an independent variable and potential moderator of attitudinal inoculation [19], or limiting it to one’s beliefs about one’s ability to protect an existing attitude toward an issue [20]. Our study, on the other hand, positioned task self-efficacy as the main target of inoculation messages. Although the absolute change displayed from pre-to-post trial by those in the treatment condition was not dramatic (i.e., a change of. 19 units on a 5-point scale), our analyses nonetheless revealed support for the viability of self-efficacy inoculation. Second, we focused inoculation in the context of a performance task (i.e., a balance activity). Previous inoculation research has typically assessed resistance to a written attack, read by individuals, or a video attack, watched by individuals. In our study, though, individuals were engaged in a performance scenario; that is, they were *doing* something, and the ‘attacker’ was physically present. Third, we provided the first examination of the potential mechanisms that may underlie self-efficacy inoculation effects. That is, we observed that individuals’ self-presentation concerns—which were lower among inoculated participants—supported an indirect relationship between the inoculation treatment and post-task self-efficacy, inasmuch as those in the inoculation condition reported lower self-presentation concerns, which subsequently aligned with more positive post-task self-efficacy appraisals.

In terms of limitations, it is worth noting that our failure to reduce control participants’ self-efficacy perceptions may have indicated that our chosen ‘attacks’ were not sufficiently severe. Accordingly, it would be interesting to determine the extent to which individuals in the control and treatment conditions may respond when faced with stronger (i.e., more frequent) verbal attacks and greater performance pressure (e.g., by amplifying the importance of the activity). In addition, participants were only provided with a relatively short period of time in which they could engage in elaborative processes in between the receipt of the message and the provision of ‘attacks’. Inoculation researchers have considered how varying the delay between inoculation treatments and subsequent attacks may impact attitude resistance [12], and it would be fascinating to begin to address similar issues with respect to the protection/promotion of task self-efficacy. Indeed, providing individuals with greater time to elaborate on refutation material may be one way through which researchers could encourage a more substantial change in self-efficacy beliefs through inoculation. It is also worth noting that we purposefully placed individuals in a novel (and controlled) environment so as to minimize the confounding effects of pre-existing knowledge structures. We are also aware that the present findings offer limited generalizability beyond undergraduate cohorts. Accordingly, it remains to be seen whether inoculation treatments may be successful in conferring resistance to relatively more entrenched self-efficacy perceptions held by individuals in diverse population groups (e.g., in relation to real-world activities, such as one’s academic or athletic pursuits). In sum, as well as representing a novel integration of the self-efficacy and persuasion literatures, these findings also offer insight into the messaging strategies that may help individuals thrive in the face of challenges to their confidence in their ability. Given that such challenges are commonplace across diverse domains of functioning, there appear to be a host of fascinating applications for the study of task self-efficacy inoculation.

## Supporting Information

S1 Appendix Inoculation treatment (i.e., counter-argument—refutation pairings). (DOCX)

S2 Appendix Alternative main analysis. (DOCX)

S1 Data Raw data file. (XLS)
