# Supplementary material for: Preempting Performance Challenges: The Effects of Inoculation Messaging on Attacks to Task Self-Efficacy
Source: PLoS One. 2015 Apr 21;10(4):e0124886. doi: 10.1371/journal.pone.0124886 (PMC4405199; doi:10.1371/journal.pone.0124886)
Supplement: S1 Appendix — (DOCX) [file pone.0124886.s001.docx]

**Appendix A: Inoculation treatment (i.e., counter-argument – refutation pairings)**

*Counter-argument – refutation pairing 1 (verbal persuasion/criticism):* “You are likely to receive criticism from the sport scientist about your performance. Don’t worry – bear in mind that sport scientists are used to dealing with highly-trained, elite athletes. They are most familiar with giving feedback to an extremely gifted population, and they do so promptly, without inhibition, and sometimes without much thought. As scientists, they tend to be focused on perfection rather than sufficiency, and they therefore have high expectations of participants in their studies.”

*Counter-argument – refutation pairing 2 (ineffective performance):* “Novel tasks are often met with mistakes. Undoubtedly you will feel that you’ve made a mistake, or even a series of mistakes. Don’t worry – there are many factors that contribute to your balance score in this task. Thousands of pieces of information are merged to obtain your overall balance score. Performance over a second or two will only have a minimal impact on the overall score achieved. Stay focused and rest assured that it is the aggregate of your performance across the task that is important.”

*Counter-argument – refutation pairing 3 (adverse emotional states):* “Given that your balance score aligns with important functional and performance indicators, it’s likely that you may feel nervous before and throughout this balance task. Don’t worry – positive or negative mood states are unlikely to affect your balance score to any substantial degree. What is felt internally will not influence your score much in a gross motor task such as this. Regardless of whether you’re feeling excited or nervous, your score shouldn’t change too much.”
