# Supplementary material for: Preempting Performance Challenges: The Effects of Inoculation Messaging on Attacks to Task Self-Efficacy
Source: PLoS One. 2015 Apr 21;10(4):e0124886. doi: 10.1371/journal.pone.0124886 (PMC4405199; doi:10.1371/journal.pone.0124886)
Supplement: S2 Appendix — (DOCX) [file pone.0124886.s002.docx]

**Appendix B: Alternative main analysis**

For our alternative main analyses, we again used a MANCOVA to investigate between-condition differences on (a) post-task self-efficacy, and (b) the length of time that participants selected for their (bogus) second trial. We again used an adjusted criterion (i.e., α = .025) when determining univariate significance, and explored differences on these two dependent variables while controlling for the potential effect of background variables (i.e., self-confidence robustness, resilience, perceived competence at agility-based tasks, pre-task tension), as well as participants’ pre-task self-efficacy and task performance score. On this occasion, all participants from the original sample were included in our analyses (excluding those who provided large amounts of missing data), irrespective of their score on the recall test (i.e., irrespective of the extent to which they attended to the message). This resulted in a usable sample of 178 participants, with 90 in the treatment group and 88 in the control group. When using the entire sample, no significant multivariate effect emerged (*F*(2, 169) = .87, *p* = .42, η^2^_p_ = .01, λ = .99), and at the univariate level, there were no differences on post-task self-efficacy (*F*(1, 170) = 1.73, *p* = .19, η^2^_p_ = .01), or the length of time that participants selected for their second trial (*F*(1, 170) = .03, *p* = .87, η^2^_p_ = .001).

It is important to consider potential interpretations for the different findings (i.e., the lack of significant effect) that we observed when exploring between-condition differences using the entire sample. On the one hand, it is possible that the effectiveness of the inoculation treatment may simply have been impaired/negated for those participants who did not elaborate on, and fully process, the message they received. Alternatively, it is also possible (and we believe likely) that those participants who scored poorly on the recall test had not actually attended to the message fully, and had failed to read the content sufficiently. The nature of the participant group (i.e., undergraduate students who simply needed to attend the lab session to receive course credit) may have resulted in a higher-than-typical level of apathy/disinterest among the sample, and this may be why we observed a relatively high proportion of individuals who simply did not read (let alone process) the message. It was for this reason that we initially sought to only include those who showed evidence that they had attended to the message. We acknowledge that the cut-off for our recall score (i.e., those who scored below the mid-point) may appear somewhat arbitrary, but we purposefully selected this cut-point so as to ensure that we did not include individuals who had correctly guessed one or two pieces of information in the recall test. The recall questions were all easily answerable to those who paid attention to the material, and so our use of those who scored at or above the mid-point effectively resulted in us restricting our investigation to a study of those who we could be certain had studied and elaborated on the message content.
